# The role of DNA methylation and demethylation in bladder cancer: a focus on therapeutic strategies

**DOI:** 10.3389/fonc.2025.1567242

**Published:** 2025-06-26

**Authors:** Wiktoria Strasenburg, Jędrzej Borowczak, Daria Piątkowska, Jakub Jóźwicki, Dariusz Grzanka

**Affiliations:** ^1^ Department of Clinical Pathomorphology, Faculty of Medicine, Collegium Medicum in Bydgoszcz, Nicolaus Copernicus University in Torun, Bydgoszcz, Poland; ^2^ Clinical Department of Oncology, Prof. Franciszek Łukaszczyk Oncology Centre, Bydgoszcz, Poland

**Keywords:** DNA methylation, DNA demethylation, TET, 5hmC, bladder urothelial carcinoma

## Abstract

DNA methylation is the best-known epigenetic mechanism regulating gene expression without altering the DNA sequence. Its counterpart, known as DNA demethylation, is equally important and enables the activation of previously silenced genes. DNA demethylation has attracted interest in the scientific community following the landmark discovery that Ten-Eleven Translocation (TET) proteins can convert 5-methylcytosine to 5-hydroxymethylcytosine. A growing body of research indicates that changes in TET protein levels and 5-hydroxymethylcytosine content are hallmarks of cancer. These epigenetic changes appear to play a critical role in the development of malignancies characterized by high levels of somatic mutations and genetic instability. Bladder cancer is among the most common cancers worldwide and, despite aggressive treatment, remains associated with high mortality and poor prognosis. The lack of reliable diagnostic and prognostic markers poses a significant challenge in its management, highlighting the urgent need for novel biomarkers to enable earlier diagnosis and more accurate prediction of clinical outcomes. This review examines epigenetic alterations associated with bladder cancer and their clinical implications. We focus on the impact of DNA methylation and demethylation on oncogene regulation, summarize scientific evidence supporting their role in bladder cancer development and progression, and briefly explore novel therapeutic strategies targeting those epigenetic mechanisms.

## 1 Introduction

Bladder cancer (BLCA) is one of the most prevalent malignancies worldwide, accounting for over 550,000 new cases and more than 200,000 deaths each year (1, 2). The number of individuals living with bladder cancer in the United States is projected to exceed 800,000 by 2030 (1). The disease predominantly affects older adults and occurs three to four times more frequently in men than in women, largely due to exposure to risk factors, such as smoking and occupational carcinogens, including aromatic amines. Among these, tobacco smoking is the most significant, contributing to over 50% of cases in men and nearly 40% in women (2). The carcinogenic effects of tobacco smoke and other exposures are mediated through both genetic and epigenetic alterations, including the accumulation of DNA methylation changes and the generation of reactive oxygen species that induce DNA damage. Other risk factors for bladder cancer include chronic bladder inflammation, such as that caused by Schistosoma haematobium infection, prolonged catheter use, pelvic radiation, and occupational exposure to chemicals, like benzidine and β-naphthylamine (2, 3). These molecular changes underlie the pathogenesis of urothelial carcinoma (UC), the predominant histologic type of bladder cancer, which accounts for over 90% of bladder cancer cases in developed countries. UC can be classified into non-muscle-invasive (NMIBC; stages Ta, T1, carcinoma *in situ* [CIS]) and muscle-invasive (MIBC; stage T2 and higher) forms (2).

Clinically, bladder cancer presents with a range of symptoms depending on the stage and extent of disease. In early-stage disease, the most common manifestation is painless hematuria, reported in up to 85% of patients. However, many early-stage tumors are asymptomatic or associated with nonspecific urinary symptoms, leading to delayed diagnosis. In advanced stages, patients may experience pelvic pain, dysuria, or systemic symptoms such as bone pain or unintentional weight loss (4).A significant challenge in BLCA management is the lack of reliable diagnostic and prognostic markers. Current methods, like urine cytology and cystoscopy are commonly used for diagnosis and monitoring; however, urine cytology lacks sensitivity in detecting low-grade tumors, while cystoscopy, an invasive procedure, still misses up to 30% of malignant cases (3). This underscores the urgent need for novel biomarkers for early cancer detection and prognosis.

Current treatment regimens vary depending on the disease stage (5). NMIBC is usually managed by transurethral resection followed by intravesical instillations of mitomycin C or Bacillus Calmette–Guérin(BCG). Radical cystectomy with or without neoadjuvant cisplatin-based chemotherapy remains the standard of care for MIBC. Metastatic disease is treated with platinum-based chemotherapy, followed by maintenance avelumab, an anti-PD-L1 agent, in patients without disease progression. Cisplatin-ineligible patients with positive PD-L1 status, as well as those who progress after platinum-based therapy, may receive atezolizumab or pembrolizumab. Targeted therapies, such as enfortumabvedotin and erdafitinib, are used in later lines and have expanded the range of available options. However, many patients develop resistance or experience treatment-related toxicity, highlighting the need for alternative approaches (5).

Over the past decade, the cancer epigenome has come to the forefront of oncogenesis, bringing significant implications for cancer initiation and progression. Epigenetics delves into heritable modifications that alter the expression of genes without changing their sequences (6). Epigenetic mechanisms, including DNA methylation and demethylation, play a key role in maintaining genome integrity. Disruption of these mechanisms caused the accumulation of genetic alterations, and leads to genetic instability, a hallmark of cancer (7, 8).

In this review, we present the impact of DNA methylation and demethylation on BLCA and discuss novel therapeutic strategies. We summarize the current state of knowledge and highlight future directions in the field.

## 2 DNA methylation

DNA methylation is one of the first known epigenetic modifications that regulates gene expression without changing its sequence. This process involves transferring a methyl group from

**Figure 1:**
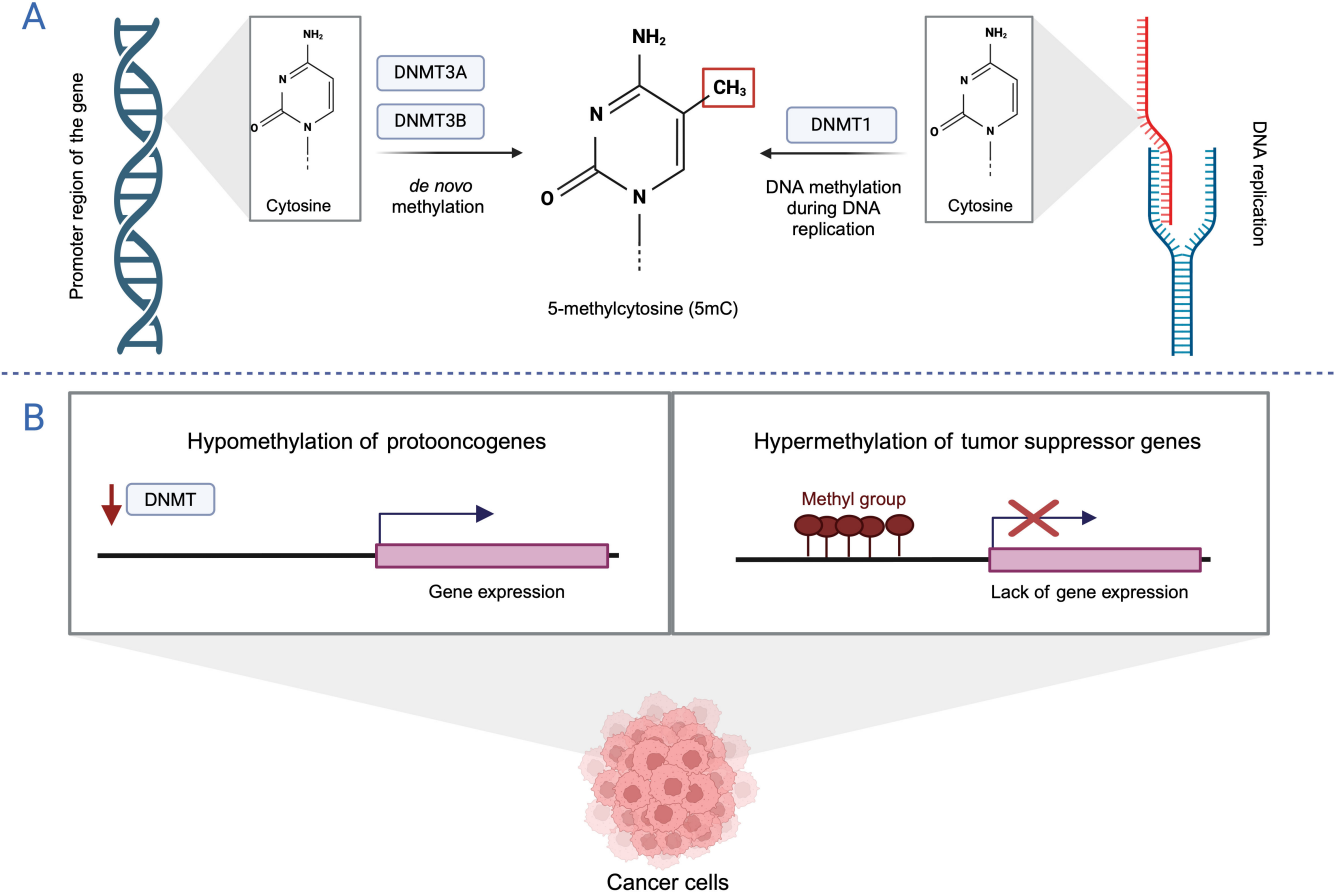
DNA methylation **(A)** DNA methylation pathways. **(B)** DNA methylation pattern in cancer. Created with BioRender.com.

S-adenosyl-L-methionine (SAM) to the 5′ position of cytosine, forming 5-methylcytosine (5-mC). DNA methylation is catalyzed by DNA methyltransferases (DNMTs), including DNMT1, DNMT3a and DNMT3b. DNMT1 preserves the methylation pattern during DNA replication. DNMT3a and DNMT3b are active methyltransferases that play a role in establishing DNA methylation patterns during early mammalian development and in germ cells (9). DNA methylation typically occurs at CpG dinucleotides, which are regions in the DNA sequence where a cytosine is followed by a guanine, separated by a single phosphate group in the 5′ to 3′ direction. In mammals, approximately 80% of CpG sites are methylated and are strongly associated with gene repression. CpG islands (CGIs), on the other hand, are genomic regions characterized by a local increase in the density of CpG dinucleotides. They are often found near promoter regions and typically remain unmethylated (10, 11).It is well known that normal epigenetic modifications are altered during the initiation and progression of tumorigenesis, leading to widespread changes in DNA methylation patterns. The cancer epigenome is characterized by global hypomethylation (with methylation levels reduced from around 80% to about 50%) and local DNA hypermethylation of CGI promoters. CGI hypermethylation is often associated with the silencing of tumor suppressor genes, genes that regulate cell growth, and downstream signaling pathways (**Figure 1**) (12). Indeed, numerous studies have identified several cancer-related genes that undergo aberrant DNA methylation in tumor cells. An abnormal methylation pattern in BLCA cells has recently been incorporated into multigene predictive models. Panels identifying specific genes like *MYO3A*, *CA10*, *NKX6-2*, and *DBC1*, which show high diagnostic accuracy for BLCA detection (13) also highlight how methylation states can serve as biomarkers. Additionally, the study by Olkhov-Mitselet et al. on the methylation differences in *GP5*, *EOMES*, and *ZSCAN12* genes between low and high-grade BLCA underscores the prognostic potential of these patterns (14).

Wolff et al. found that non-muscle invasive BLCA and muscle invasive BLCA follow distinct epigenetic pathways in tumorigenesis. The study revealed that invasive tumors exhibited widespread hypermethylation, while non-invasive tumors exhibited hypomethylation. The *IPF1*, *GALR1*, *TAL1*, *PENK*, and *TJP2* genes were significantly hypermethylated in tumor-derived samples compared to both cancer-free urothelial samples and corresponding normal-appearing tissues. Sequencing analysis confirmed the absence of abnormal DNA methylation patterns in the urothelium of a BLCA-free patient, with low levels of DNA methylation observed in normal-appearing tissue. In contrast, the urothelial tumor from the same patient exhibited a high level of DNA methylation. These results suggest that epigenetic alterations are early events in bladder carcinogenesis and that changes in methylation patterns can be detected in apparently normal cells before tumorigenesis occurs. These findings have important implications for the earlier detection of BLCA and the identification of patients at increased risk for recurrence. A substantial body of research supports the use of disease-related methylation changes as biomarkers for various diseases, including BLCA (15).

Bladder EpiCheck is a post-treatment monitoring tool designed to detect the recurrence of NMIBC in patients with a prior diagnosis. This approach has the potential to reduce the frequency of follow-up cystoscopies. The test analyzes 15 methylation biomarkers in urine to identify BLCA. In a study involving 353 patients the test effectively rules out high-grade tumors, with a negative predictive value of 99.3% and shows a sensitivity of 91.7% for detecting their presence. The authors conclude that the test can help reduce unnecessary cystoscopy procedures in the follow-up of NMIBC, as it reliably detects high-grade recurrences. By alternating cystoscopy and cytology with Bladder EpiCheck, the burden of these procedures could be significantly reduced (16).

The precise mechanisms underlying the reduction of DNA methylation in cancer cells remain poorly understood. One proposed explanation is the involvement of TET proteins, essential enzymes that mediate active DNA demethylation. Alterations in TET activity and function have been linked to malignant transformation. Understanding how TET proteins and their regulators affect cancer development may provide valuable insights into potential therapeutic strategies and diagnostics.

## 3 DNA demethylation

Since 2009, DNA demethylation has attracted considerable attention due to the discovery that Ten-Eleven-Translocation (TET) proteins convert 5mC to 5-hydroxymethylcytosine (5-hmC) (17). Two distinct pathways for demethylation have been identified: active demethylation and passive demethylation.

**Figure 2:**
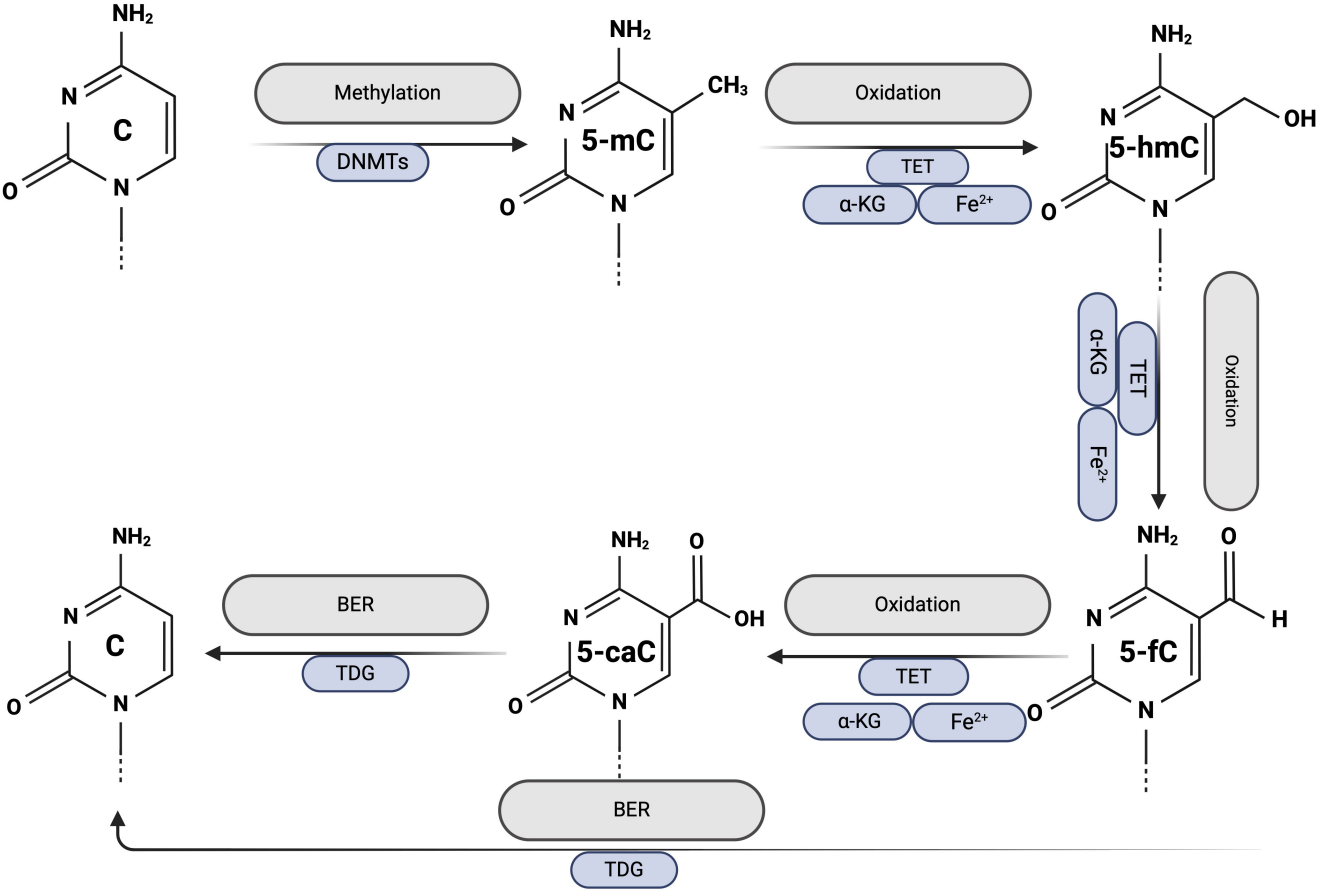
Active DNA demethylation. Created with BioRender.com.

Active DNA demethylation can occur in both dividing and nondividing cells, is initiated by the TET enzymes (TET1, TET2, TET3) and requires α-ketoglutarate (αKG) as a co-substrate and Fe2+ and vitamin C as co-factors; therefore, TET activity depends on the availability of these factors. The first step of active demethylation involves the gradual oxidation of 5-mC to 5-hmC, 5-formylcytosine (5-fC), and 5-carboxylcytosine (5-caC). Subsequently, 5-fC and 5-caC are recognized by the thymine DNA glycosylase (TDG), which activates the base excision repair pathway (BER) and leads to the replacement of the modified cytosine with an unmodified cytosine (18). The exact mechanism by which TET enzymes convert 5-mC to unmethylated cytosine during active DNA demethylation is shown in **Figure 2**.

Passive DNA demethylation occurs in dividing cells. The maintenance of previously established DNA methylation patterns requires continuous methylation of hemimethylated DNA, a process ensured by the activity of DNMTs during DNA replication. Inhibition or dysfunction of DNMTs allows newly synthesized cytosines to remain unmethylated, leading to a gradual reduction in overall DNA methylation levels with each round of cell division (19).

### 3.1 DNA demethylation: 5-hydroxymethylcytosine

5-hmC is a stable modification with much higher levels in the genome than its oxidized derivatives (20). 5-hmC is not only an intermediate of passive and active DNA demethylation but also partakes in the regulation of gene expression through association with various epigenetic regulators (21, 22). The level of 5-hmC is relatively low in all cells, but the distribution of 5-hmC is cell type-dependent and differs between specific tissues. For instance, its levels are higher in the central nervous system (CNS) and embryonic stem cells (ESCs) compared to peripheral tissues (23, 24). The highest levels of 5-hmC were found in post-mitotic cells, especially within the CNS, and significantly lower or undetectable levels in proliferating cells (except ESCs) (24–26).Extremely reduced levels of 5-hmC were detected in highly proliferating cancer cells and may contribute to worse patient prognosis (25).Despite numerous studies on cancer epigenetics, the precise role and mechanism of 5-hmC in bladder tumorigenesis remain unclear. We aim to highlight recent reports exploring the involvement of 5-hmC in BLCA and its impact on patient prognosis.

Peng et al. explored the role of 5-hmC in BLCA and characterized its association with tumorigenesis, progression, and patient outcomes. They used immunohistochemistry (IHC) to compare the levels of 5-hmC in matched BLCA tissue (n=135) and normal bladder tissue (n=135).

5-hmC levels were significantly lower in BLCA than in normal bladder tissue. Moreover, patients with partial or complete loss of 5-hmC had shorter overall survival (OS), higher tumor stage, and lymph node metastases. The 5-hmC content was also reduced in BLCA cells (T24, 5637, UMUC-3, and J82) compared to controls (SV-HUC-1 and Hum-u007). Using a hydroxymethylated DNA immunoprecipitation (hMeDIP) approach coupled with deep sequencing (hMeDIP-seq), the authors found that 5-hmC is enriched in gene-rich regions in normal bladder genome and is relatively low in the BLCA genome. They mapped the hypomethylated genes revealing their close association with cancer-related pathways. The authors’ findings indicate that the loss of 5-hmC represents a novel hallmark of BLCA, with significant implications for prognosis and patient outcomes (27).

Munari et al. investigated the cancer-specific loss and distribution of 5-hmC in bladder urothelial cell carcinoma. They demonstrated that 5-hmC nuclear staining levels in BLCA are lower than in normal bladder tissue. However, this study showed no direct association between 5-hmC distribution patterns, cancer cell proliferation index measured by Ki-67 staining, and clinicopathological features of BLCA or patients’ prognosis (28). The results of this study contrast reports where 5-hmC levels were negatively correlated with tumor grade and showed prognostic significance (29, 30). However, the authors acknowledged that the overall number of progression and disease-specific death events was low, so the study was underpowered to detect small prognostic differences. Moreover, 5-hmC levels did not vary between invasive and non-invasive carcinomas, suggesting that loss of 5-hmC may occur early in bladder carcinogenesis. Notably, changes in 5-hmC levels were restricted to BLCA cells, whereas tumor-associated stroma and adjacent benign bladder tissue showed robustly high 5-hmC levels, suggesting that low 5-hmC may be useful as a biomarker for cancer detection (28).

Qi et al. investigated changes in 5-hmC during genitourinary (GU) carcinogenesis, focusing on prostate, urothelial, and renal cancers. They confirmed the presence of tissue-specific 5-hmC patterns in both healthy and cancerous GU tissues. Their findings revealed that normal kidney tissues exhibited the highest levels of 5-hmC, while normal urothelial tissues showed the lowest 5-hmC levels at the gene body. Furthermore, the analysis revealed a significant loss of 5-hmC in all three types of GU cancers compared to their matched normal tissues. Specifically, they observed a consistent pattern of hydroxymethylation at both the promoter and gene body of *HOXB8* and *HOXB9* in normal GU tissues. The promoter of the oncogene *WNT7B* was uniformly hydroxymethylated in GU cancers but not in normal tissues. Additionally, they demonstrated that both gain and loss of 5-hmC occurred during GU tumorigenesis. Genes with increased 5-hmC levels were predominantly involved in pathways related to stemness, hypoxia, and immunity, while genes with decreased 5-hmC levels were associated with pathways related to proliferation, metabolism, and cell adhesion. Overall, these findings suggest that 5-hmC alterations may serve as a hallmark of GU tumorigenesis (31).

The cancer epigenome is characterized by global hypomethylation with locus-specific hypermethylation. Variation in methylation patterns can inactivate tumor suppressor genes and activate protooncogenes, a hallmark of cancer. Bladder cancer is characterized by a global loss of 5-hmC, suggesting an important role in cancer development and progression. Several mechanisms may underlie 5-hmC depletion in cancer, including mutation of TET enzymes, reduction of TET activity by hypoxia, or IDHs mutations. It seems important that only a few reports show an association with clinical and pathological features. There is an urgent need for further studies to explore the prognostic value of this marker.

### 3.2 DNA demethylation: TET family of enzymes

**Figure 3:**
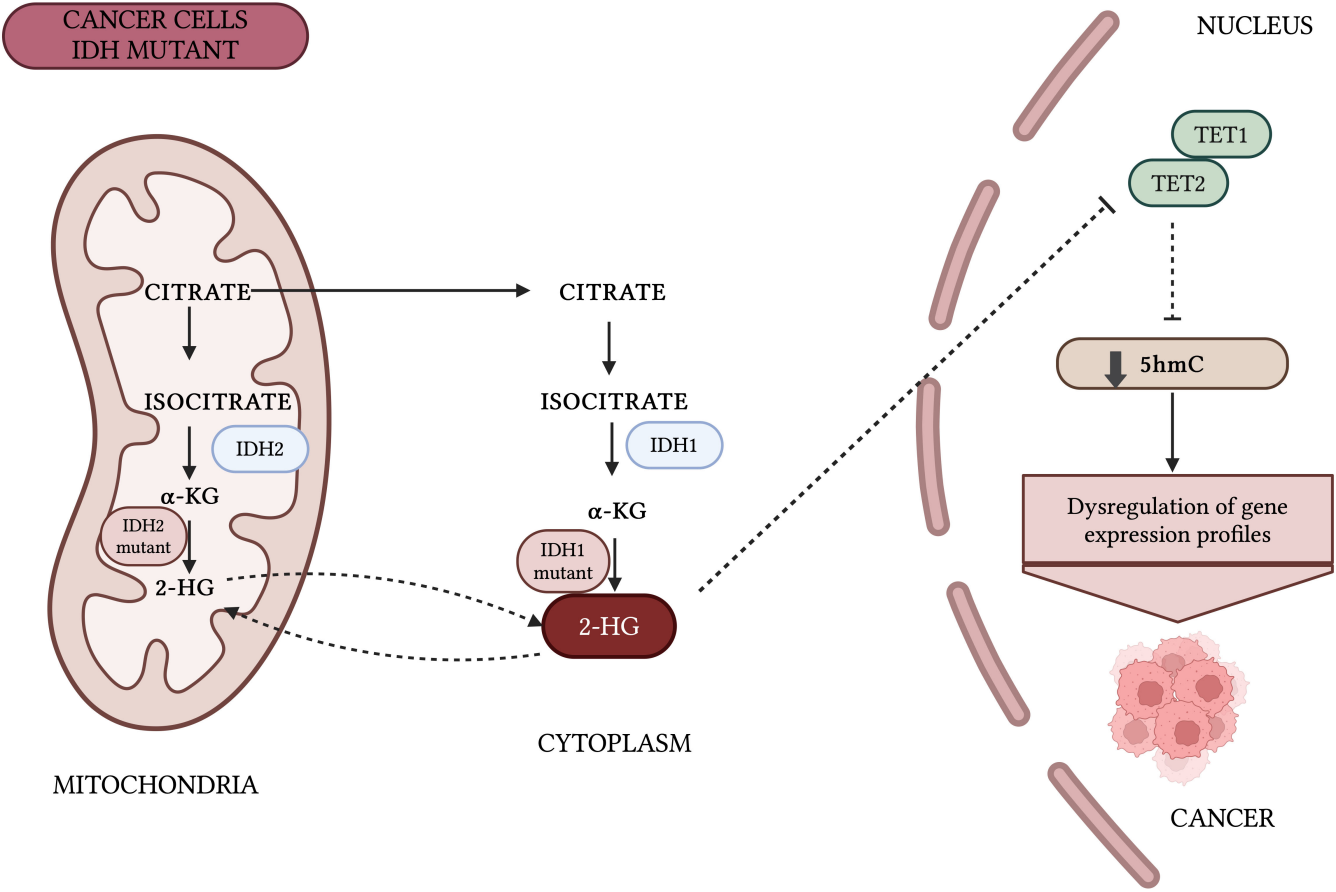
Impact of IDH mutations on DNA methylation patterns. Mutant IDH1/2 generates oncometabolite 2-hydroxyglutarate (2-HG) instead of α-KG, which inhibits the enzymatic activity of TET proteins. Thus, IDH1/2 play an important role in the regulation of 5-hmC. Inhibition of TET enzymes by 2-HG leads to hypermethylation of CGIs at gene promoters, disturbs cell differentiation, and promote cancer transformation (36, 89).Created with BioRender.com.

There are three members of the TET family of enzymes, TET1, TET2, and TET3, which act as iron(II)/α-ketoglutarate (Fe(II)/α-KG)-dependent dioxygenases. TET proteins play a central role in DNA demethylation, catalyzing the sequential oxidation of 5-mC to 5-hmC, 5-fC, and 5-CaC (17, 32). Research increasingly shows the importance of TET-mediated 5-mC oxidation in health and diseases, including cancers. Dysfunctions of TET proteins, often due to mutations or aberrant expression of their regulators, are common in hematological disorders and less frequent in solid cancers. This suggests other mechanisms are likely responsible for the frequent loss of 5-hmC in solid tumors (33–35).The enzymatic activity of TET strongly depends on the availability of Fe2+ as a reaction cofactor and αKG as a co-substrate. αKG is generated from isocitrate in the citric acid cycle catalyzed by the isocitrate dehydrogenase (IDH). Alterations in DNA methylation resulting from IDH mutations are common in a wide range of cancers, including but not limited to acute myeloid leukemia, glioma, and cholangiocarcinoma (**Figure 3**) (36–39).

Here, we summarize several studies on TET proteins and their involvement in bladder tumorigenesis. It is important to emphasize that data on TET proteins in BLCA are limited, despite their well-established role in other cancers.

Zhu et al. identified a new signaling pathway that may be responsible for the development of invasive BLCA. Although the study does not directly address the topic of epigenetics, the findings suggest that CD44s deregulation may be linked to the TET protein. The authors revealed that overexpression of autophagy-related gene 7 (ATG7) promotes the degradation of AU-rich element RNA-binding protein 1 (AUF1), which stabilizes *TET1* mRNA. Upregulation of TET1 contributes to the demethylation of the ubiquitin-specific peptidase 28 (USP28) promoter, increasing USP28 protein expression and leading to the accumulation of CD44s protein. CD44s inhibits RhoGDIβ degradation, which in turn promotes the development of invasive BLCA and lung metastasis. The interplay between ATG7, and its potential impact on the stability and function of TET proteins represents a promising avenue for future investigation (40).

Hu et al. reported a novel XIST-TET1-p53 regulatory network in BLCA cells that affects cell proliferation, migration, and apoptosis. The authors performed RNA immunoprecipitation (RIP) and chromatin immunoprecipitation (CHIP) assays in T24 and 5637 cells. The RIP experiment showed that X-inactive specific transcript (XIST) can bind to TET1. XIST knockdown in the T24 cell line significantly upregulated *TET1* and *p53* promoter binding levels, whereas overexpression of XIST in the 5637 cell line significantly downregulated *TET1* and *p53* promoter binding levels. The results demonstrated that TET1 can promote p53 expression by binding to the promoter region of *p53*, while XIST inhibits p53 expression by binding to *TET1*. These findings suggest that XIST acts as an epigenetic regulator, directly interacting with TET proteins and affecting their functions in BLCA (41).

Yan et al. reported that TET1 expression was downregulated in BLCA samples compared to matched adjacent normal bladder tissue and was inversely associated with tumor stage and overall survival. The authors also demonstrated that adherens junction-associated protein 1 (AJAP1) was downregulated in BLCA tissue, particularly in muscle-invasive BLCA specimens, compared to non-muscle-invasive BLCA specimens. AJAP1 levels negatively correlated with T stage and grade, and patients with low *AJAP1* mRNA levels had shorter overall survival. To confirm that AJAP1 can be silenced by DNA methylation, the researchers treated T24 and J82 cell lines with the DNA methylation inhibitor 5-aza-dC. After treatment, *AJAP1* expression was significantly increased in both cell lines, suggesting that *AJAP1* is regulated by promoter methylation. Furthermore, they found that TET1 maintained the hypomethylation in the *AJAP1* gene promoter. Downregulation of TET1 in BLCA cells failed to maintain *AJAP1* expression, leading to the activation of the Wnt/β-catenin signaling pathway. These data suggest that TET1 suppresses BLCA cell growth through regulation of the β-catenin signaling pathway (42).

TET proteins may function as tumor suppressor genes, playing a role in many signaling pathways associated with bladder carcinogenesis. While current studies have revealed their interactions with proteins and genes such as *CD44s*, *XIST* and *AJAP1*, the full scope of TET’s functions in BLCA remains unclear. Further research is needed to better understand these mechanisms and the potential of TET as a therapeutic target.

## 4 Targeting DNA methylation in BLCA

While global hypomethylation of epigenome facilitates the acquisition of somatic mutation and is a landmark of BLCA, hypermethylation is a local event that affects predominantly the promoter regions of suppressor genes. It occurs early during BLCA pathogenesis and enables cancer cells to bypass cell cycle control and evade apoptosis (12, 43). Furthermore, as the disease progresses from non-muscle invasive to muscle invasive BLCA, the prevalence of the methylation pattern increases, indicating its role in aggravating cancer clinical course (15). As such, drugs demethylating and reactivating the promoter regions of suppressor genes or preventing their methylation emerged as a promising therapeutic approach, with DNMT inhibitors constituting the most frequently used type of drugs.

However, despite significant advances in the epigenetics of BLCA, the armamentarium of available therapeutic agents is limited. While BLCA patients are routinely treated with intravesical BCG, intravesical chemotherapy, or cisplatin-based chemotherapy, depending on the stage of the disease, no DNMT-targeted strategy is currently used as a standard treatment approach. Furthermore, most agents showed varied degrees of specificity to DNMTs. Nucleoside analogues, such as 5-azacitidine and decitabine have been approved by the FDA for the treatment of multiple hematological malignancies, but their use is associated with high toxicity related to low specificity to DNMT (44, 45). Non-nucleoside analogues inhibit DNMT independently of DNA incorporation, but bind to DNA, compete for its binding sites of DNMTs, and seem to cause less side effects (46).

While DNMT inhibitors became a potential treatment modality for BLCA, they have yet to enter phase III clinical trials. Furthermore, despite the rapid development of novel drugs, most recent studies have focused on adapting DNMT inhibitors with proven clinical efficacy in other malignancies to BLCA. Particular attention has been paid to studies incorporating DNMT inhibitors into other therapeutic regimens and aiming to reduce therapy-associated toxicities. Below, we organized recent reports regarding studies targeting DNA methylation in BLCA and discussed their potential use in clinical practice (**Supplementary Table 1**).

### 4.1 5-azacitidine

5-azacitidine (5-Aza) is a cytidine analog and a ribonucleotide whose incorporation into RNA disassembles polyribosomes, disturbs the functions of starter RNA, and stops protein production. Although to a smaller extent, 5-Aza is also incorporated into DNA and covalently binds to DNMTs, preventing DNA synthesis. It evokes a dose-dependent antineoplastic effect in two ways: at lower doses, 5-aza includes DNA hypomethylation by inhibiting DNMT1 activity while causing direct cytotoxicity at high doses (47).

In preclinical settings, 5-Aza inhibited the proliferation of BLCA cells, suppressed cancer growth in mice, and increased the susceptibility to cisplatin and docetaxel in T24 cells (48–50). However, the combination of 5-Aza and phenylbutyrate lacked clinical efficacy in patients with locally advanced and metastatic BLCA (51). In recent phase II clinical trials, the overall response rate to 5-Aza in patients with advanced solid tumors reached 4.8% (3/62) and 12.5% (1/5) in patients receiving 5-Aza, pembrolizumab, and epacadostat. No data regarding patient results in BLCA alone were posted (52).

Two phase I clinical trials assessing the safety of CC-486, an oral analog of 5-azacitidine, were completed in 2018. Von Hoff et al. reported that CC-486 was well tolerated by patients either in monotherapy or with carboplatin. Of 22 BLCA patients, 3 (13.6%) achieved partial remission, and the disease control rate was 36.4% (53). The results of the second trial have not yet been published (54).

### 4.2 FdCyd

5-fluoro-2′-deoxycytidine (FdCyd) is a fluoropyrimidine nucleoside analog that is phosphorylated and incorporated into DNA, binding to DNMT and inhibiting DNA methylation. Its rapid metabolism produces 5-fluoro-2′-deoxyuridine (FdUrd), a cytotoxic inhibitor of DNA replication. FdCyd, unlike other cytidine analogs, is stable in aqueous solutions (55, 56). In the recent phase II clinical trial, 18 patients with urothelial carcinoma received FdCyd with tetrahydrouridine (THU). One patient (5.6%) achieved partial response, the median PFS was 3.6 months, and the 4-month PFS probability reached 42%. The treatment was well tolerated, but the strata were terminated preemptively due to slow accrual (55).

### 4.3 Decitabine

Decitabine (5-aza-2’-deoxycytidine) is a DNMT1 inhibitor, which, as a deoxyribonucleoside, can be incorporated into DNA and affect its methylation (47). While decitabine is widely used to treat leukemia, its potential use in BLCA is still under investigation (57).

Preclinical studies showed that decitabine restores the methylation landscape in BLCA (58). Multiple genes responsible for antitumor and inflammatory response, such as *p53*, *NOTCH1*, and *p73*, were hypermethylated in BLCA, and their expression increased after treatment (59, 60). Those effects seemed dose-dependent and resulted in the inhibition of proliferation, migration, and invasion and enhanced apoptosis of cancer cells (61–63). Decitabine demethylated the *p16* gene and reactivated p16 activity, thus restoring cellular anticancer response. In T24 cells, changes resulting from p16 reactivation, such as cell growth inhibition and G1 cell cycle, persisted after cell division (64). Other studies proved that those changes were associated with the inhibition of DNMT1 and DNMT3b and the downregulation of their mRNA transcript. Since *de novo* methylation of CGIs occurs in dividing cells, DNA-demethylating agents require DNA replication to be effective. However, the precise relationship between their efficacy and proliferation rate and therapeutic efficacy remains to be fully elucidated (65).

Decitabine increased cancer cells’ susceptibility to other therapeutics and changed the activity of pathways traditionally associated with carcinogenesis. Low doses of decitabine enhanced the efficacy of cisplatin and gemcitabine *in vitro* and *in vivo* (66). Mechanistically, the treatment increased caspase and the number of cells entering subG1 and G2/M phases independently of p53 (67). The inhibition of BLCA stemness may also occur via the activation of interferon signaling and the suppression of ERK and STAT3 pathways (66, 68, 69). Decitabine increased the efficacy of cisplatin and gemcitabine in basal-like BLCA cells and mice, while the combination of gemcitabine, cisplatin, decitabine, and trichostatinA (TSA) downregulated *c-Myc* and *cyclin D1* genes and the expression of the antiapoptotic BCL2L1 (66, 70). Pre-treatment with decitabine and a histone deacetylase inhibitor enhanced BLCA cells’ sensitivity to cisplatin. Still, this effect was cell line-specific and lacked efficacy in the 97–1 cisplatin-resistant cell line (71). Those results aligned with other studies describing increased efficacy of cisplatin and doxorubicin when combined with decitabine (60, 72). Wang et al. reported that while neither cisplatin with entinostat, another histone deacetylase inhibitor, nor decitabine could alleviate chemoresistance, decitabine with entinostat synergistically induced apoptosis and cell cycle arrest in BLCA cells (73).

Despite multiple preclinical studies, we found only two clinical trials investigating the use of decitabine in BLCA. Kassouf et al. reported that ten patients with advanced solid tumors were treated with decitabine and genistein during a phase I/II clinical trial, and 5 of them (50%) achieved stable disease. Among them were patients with BLCA (74). The results of the second study, a phase I clinical trial conducted in 2008, are still unavailable (75). The ability of genistein to modify the activity of DNMT was recently investigated to reduce the side effects of BCG treatment in superficial BLCA. Genistein showed modest improvement in symptoms with no significant differences in recurrence rates compared to controls. However, the trial failed to meet the projected enrolment and its statistical power is limited by small sample size (76).

### 4.4 Aza-T-dCyd

5-Aza-4’-thio-2’-deoxycytidine (aza-T-dCyd) is a nucleoside analog and a novel, orally bioavailable DNMT1 inhibitor (77). Compared to 5-Aza and decitabine, aza-T-dCyd more selectively depletes DNMT1, allowing for effective therapy while limiting drug-associated toxicities (78). Aza-T-dCyd showed antitumor activity alone and in combination with THU in leukemia-bearing mice, which was attributed to the simultaneous inhibition of DNMT1 and DNMT3B (79). In BLCA patients, xenograft aza-T-dCyd delayed tumor growth, inhibited DNMT1 expression, and upregulated the expression of tumor suppressor *p21* (80).

### 4.5 Guadecitabine

Guadecitabine (or S110) is a second-generation dinucleotide of decitabine and deoxyguanosine. As a prodrug, it is metabolized to decitabine and has a longer half-life and activity time than intravenous (81). Upon activation, guadecitabine inhibits DNMT1 and causes non-specific DNA hypomethylation (82). Combined with anti-PD-L1 and CTLA-4 agents, guadecitabine reduced the growth and metastases in the B16F10 murine melanoma model, which occurred via enhancing effector memory CD8+ T cells and spleen NK cells while also decreasing the activity of cancer-associated lymphocytes in the tumor microenvironment (81).

In T24 cells, guadecitabine caused dose-dependent demethylation at concentrations similar to 5-Aza, but the effect decreased when the dose exceeded 10 μmol/L (83). Guadecitabine demethylated the *p16* promoter, increasing its expression and stopping mouse tumor growth. The treatment was also better tolerated than 5-Aza (84). In patients with locally advanced, non-metastatic BLCA, guadecitabine reduced the dose intensity of gemcitabine without causing excessive toxicities or compromising radical treatment options post-chemotherapy (85). In a recent phase II clinical trial, guadecitabine with atezolizumab, an anti-PD-L1 antibody, failed to achieve a clinical response. However, the treatment was associated with longer survival (86).

**Figure 4:**
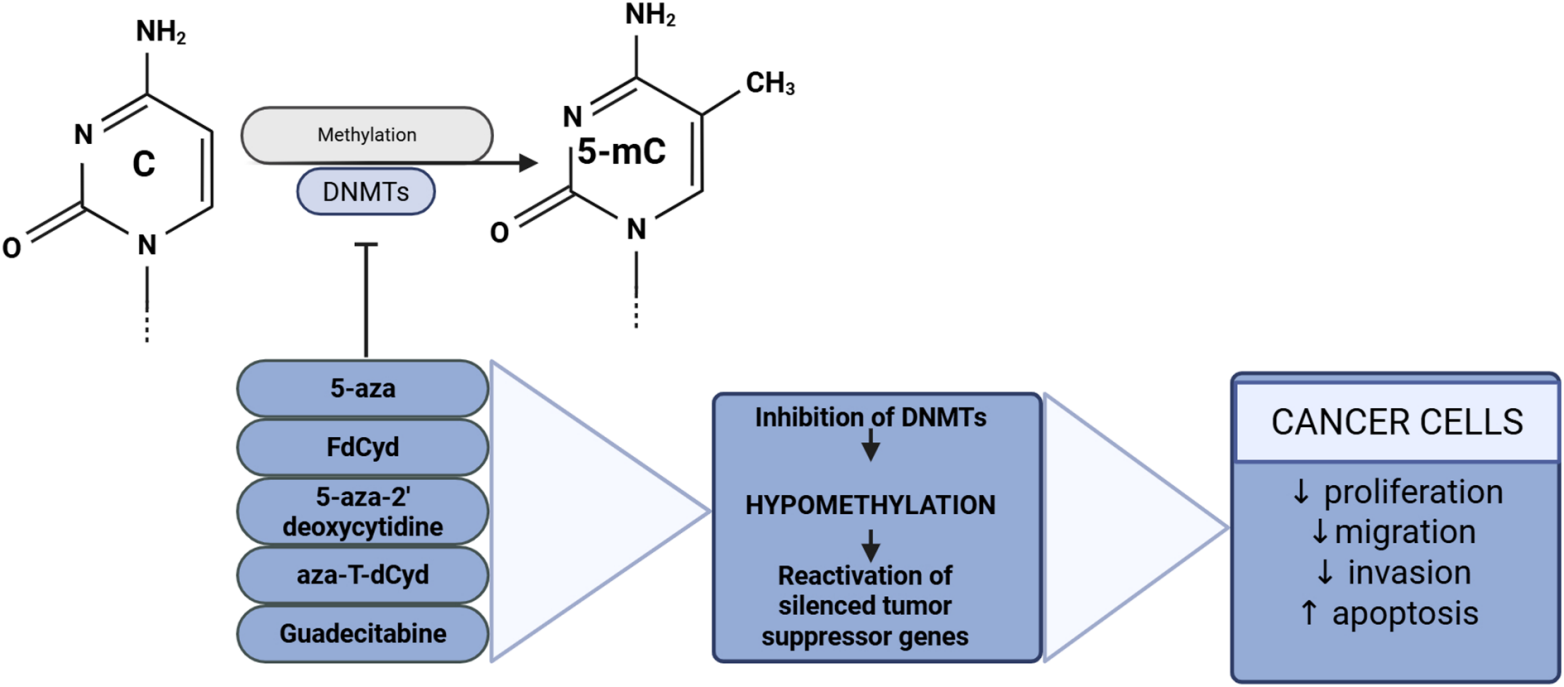
DNA methyltransferase inhibitors and their effects on cancer cells. Created with BioRender.com.

An overview of treatment-induced DNMT inhibition in cancer cells is presented in **Figure 4**.

### 4.6 TET-targeting therapies

The expression of immune checkpoints and their receptors is extensively modulated by epigenetic mechanisms. Preclinical studies have shown that loss of TET function enables cancer cells to evade antitumor immunity and resist anti–PD-L1 therapy. In colon and melanoma tumors, TET2 deletion leads to reduced chemokine expression and decreased tumor-infiltrating lymphocytes, facilitating immune evasion and therapy resistance. Furthermore, vitamin C can stimulate TET activity, increasing chemokine expression and lymphocyte infiltration, which enhances antitumor immunity and improves the efficacy of anti–PD-L1 treatment (87). Similar findings were reported in another study, which demonstrated that vitamin C enhances immunotherapy efficacy, while loss of TET2 function enables renal cell carcinoma cells to evade antitumor immunity and resist anti–PD-L1 therapy (88). While these effects have been demonstrated in multiple cancer models, research specifically focusing on bladder cancer remains limited.

Vitamin C, a well-established cofactor for iron- and α-ketoglutarate-dependent dioxygenase enzymes, plays a key role in the regulation of TET enzymatic activity. Numerous studies exploring its role in epigenetic regulation have demonstrated that vitamin C enhances the enzymatic activity of TET proteins. Mechanistically, vitamin C directly interacts with the catalytic domain of TET proteins, thereby promoting their enzymatic function. Preclinical studies have demonstrated that vitamin C treatment can reduce malignant phenotypes of bladder cancer both *in vitro* and *in vivo* by increasing the global levels of 5hmC (25, 39). A single-arm, two-stage phase I/II trial evaluated the safety and efficacy of intravenous vitamin C (IVC) combined with gemcitabine and carboplatin as neoadjuvant therapy in cisplatin-ineligible muscle-invasive bladder cancer. Twelve patients received one cycle of carboplatin plus IVC over 21 days, followed by cystectomy within 4–6 weeks. Pathological downstaging was observed in 4 patients (36%), including 3 complete responses, one of which occurred in a patient with the plasmacytoid variant. Treatment was well tolerated with minimal adverse events. The trial met criteria to proceed to stage two (87). Although the current clinical evidence is limited, these promising findings suggest that TET modulation via vitamin C could represent a novel therapeutic approach for bladder cancer, warranting further investigation.

Alterations in DNA methylation caused by mutations in IDH1 and IDH2 are common in a wide range of cancers. Mutant IDH1/2 enzymes produce the oncometabolite 2-hydroxyglutarate (2-HG) instead of α-KG. Due to its structural similarity to α-KG, 2-HG inhibits the enzymatic activity of TET proteins (36, 89). Thus, IDH1/2 may play an important role in the regulation of 5-hmC. Inhibition of TET enzymes by 2-HG leads to hypermethylation of CpG islands at gene promoters, disturbs cell differentiation, and may promote cancer transformation (38, 39, 90, 91). FDA-approved drugs such as ivosidenib and enasidenib, which target IDH1 and IDH2 respectively, have shown clinical efficacy in treating IDH-mutant AML, glioma, and cholangiocarcinoma, both as monotherapies and in combination therapies (88, 92–95). There are currently no scientific reports investigating the use of TET protein inhibitors in bladder cancer treatment. However, since the catalytic activity of TET enzymes plays a crucial role in suppressing oncogenesis in bladder cancer, identifying agents that restore TET function could offer a promising avenue for the development of novel epigenetic cancer therapies

### 4.7 Emerging therapeutics

Most of the above drugs have already been studied and used to treat other malignancies. Nevertheless, the recent advances in manufacturing technology has caused the emergence of novel drug modalities that can soon expand our treatment arsenal.

Zebularine is a novel DNMT inhibitor and a cytidine analog characterized by low toxicity in mice, even after prolonged administration (96). In BLCA T24 cells, zebularine completely depleted DNMT1 and decreased the expression of DNMT3a and DNMT3b3 (97). It prolonged the doubling time of BLCA cells to a similar degree to decitabine, but the effect was cell-line specific (98).

CM-272 is a dual-target quinolone inhibitor of G9a histone-methyltransferase and DNMT1. High G9a expression was associated with poor outcomes in BLCA, and targeting its activity was proposed as a potential treatment strategy. Its efficacy improved when used with anti-PD-L1 agents to treat BLCA in mice (99). Since the efficacy of CM-272 was limited in a hypoxic environment, Liu et al. designed a Fe3+-based nanoscale metal-organic framework (MIL-53) to accelerate the release of CM-272 and improve its clinical utility (100). This study paved the way to alleviate BLCA resistance to therapy.

Novel DNMT inhibitors are undergoing clinical trials to test their efficacy in BLCA. The safety profile of RX-3117, a cytidine analog and a DNMT1 inhibitor, was recently evaluated in a phase I/II clinical trial. Among patients taking 700mg of RX-3117 five times a week, only 10% (8/10) had adverse events associated with treatment. 92 out of 114 patients (72%) had disease progression; the best overall response in the BLCA cohort reached 45.2%, but no patients completed phase II (101). The results of another phase I study investigating the use of NTX-301, an oral DNMT1 inhibitor, with platinum-based chemotherapy in patients with advanced BLCA, are yet to be published (102).

Many preclinical studies utilize short-hairpinRNAs (shRNA) to investigate the role of the target. For instance, shDNMT3b, a shRNA targeting DNMT3b, reduced the levels of miR-461 promoters, reducing the aggressiveness of cancer cells. Similarly, shDNMT3b downregulated miR-34a, inhibiting epithelial-mesenchymal transition and migration of BLCA cells (103). While clinical application of shDNMTs seems unlikely, they can advance our understanding of DNMTs biology and the development of their inhibitors. In 2020, Liu et al. used shDNMT1–1 to investigate the role of miR-152 and developed a miR-152 mimic that inhibited the proliferation and migration of BLCA cells (104).

## 5 Perspectives and limitations

Epigenetic therapies hold significant promise, but their application in solid tumors, such as BLCA, faces substantial challenges. This section outlines key limitations of current approaches and highlights strategies that may enhance their utility.

DNMT inhibitors have demonstrateda broad spectrum of anticancer activity in both *in vitro* and *in vivo* studies, but data regarding their use in BLCA are predominantly from preclinical studies. The traditional DNMT inhibitors, especially 5-azacitidine and decitabine, have shown great clinical efficacy in hematologic malignancies, but lacked efficacy when used alongside immunotherapy in BLCA. It appears that the complexity of the bladder cancer tumor microenvironment, frequent occurrence of hypoxia, dense stromal components, and intrinsic mechanisms of therapeutic resistance complicate the clinical translation of epigenetic therapies (105–107).For example, 5-aza with sodium phenylbutyrate lacked clinical efficacy in locally advanced tumors, while the combination of decitabine and genistein achieved only modest activity in advanced solid tumors (51, 74). This is in line with broader observations that epigenetic approaches have achieved higher efficacy in hematological malignancies due to the lower intratumor heterogeneity, greater reliance on epigenetic dysregulation as a driver mechanism and better drug accessibility (108). On the other hand, the addition of 5-aza and decitabine to other therapeutic regimens increased the sensitivity of cancer cells to therapy and increased the efficacy of cisplatin, doxorubicin, and docetaxel (49, 72).

A number of factors may undermine the transition from preclinical models to clinical settings. Cell lines and animal models often fail to reflect the complexity of tumor microenvironment and heterogeneity of the urothelial carcinoma. Furthermore, models like NOD/SCID mice are commonly transected with human cancer cells but lack the key components of the immune response system, such as T and B lymphocytes (109). Hence, preclinical trials do not accurately demonstrate the clinical efficacy of anticancer agents. This shortcoming is not limited to the host immune system. Mouse models rarely reflect the crosstalk within tumor immune microenvironment and drug distribution within its stroma and into tumor cells (110). Drug efficacy is also influenced by its pharmacokinetics and metabolism, which may differ significantly between mice and human subjects. Finally, tumors in mouse models derive from a relatively homogenous population of cells collected from human malignancies or specific cell lines (109, 111, 112). BLCAs found in humans are highly heterogenous, hence specific subsets of cancer cells vary in gene expression and sensitivity to therapy (113, 114). Those factors may contribute to the frequent lack of efficacy of epigenetic agents in humans, highlighting the discrepancy between preclinical and clinical trials.

A new generation of DNMT inhibitors is emerging; however, their clinical utility remains to be proven. Guadecitabine failed to increase the efficacy of atezolizumab in severely ill patients resistant to PD-L1 therapy, but the low susceptibility to treatment in these patients makes drawing conclusions challenging (86). Conversely, RX-3317 demonstrated an overall response rate of 45.2% in metastatic BLCA, indicating potential for further investigation (101). Unfortunately, the current arsenal of anti-methylating agents is limited primarily to DNMT inhibitors and a few agents from other categories. Despite the importance of TET proteins in the control and maintenance of DNA methylation, no TET inhibitors or activators have been developed. Additionally, hypotheses regarding the potential efficacy of other agents are based on preclinical studies, which currently lack sufficient justification to enter next phases of clinical trials.

The translation from research to clinical settings would require their incorporation into the modus of precision oncology. This process will likely consist of several steps. Firstly, the testing of epigenetic drugs will likely benefit or even require predictive biomarkers for specific epigenetic signatures. Hence, biomarkers for patient selection will also be needed (108, 115). Due to rapid acquisition of therapy resistance, multipathway targeting with combination therapy—including for example DNMT inhibitors, immune checkpoint inhibitors, and PARP inhibitors — is likely to become the standard in clinical trials (116). Furthermore, combined use of low doses of HDAC inhibitors and DNMT inhibitors might alleviate chemoresistance by reversing epigenetic alterations that drive the resistance phenotype (117–119).

Although epigenetic therapies continue to attract research interest, their clinical impact in bladder cancer remains limited. Most available data derive from preclinical studies, and while some trials have provided signals of anticancer activity, they have failed to demonstrate robust efficacy in patients. Preclinical outcomes, such as chemosensitization and modulation of immune response, have yet to show clinical benefit. Moreover, other strategies, such as employing artificial intelligence models in drug development, integration of epigenetic data, 3D genome modeling, and identification of synergistic drug combinations for further trials might enhance the effectiveness of such therapeutic regimens (120, 121). Going forward, the development of biomarker-driven strategies and combination approaches will be critical to overcoming current limitations and realizing the potential of epigenetic modulation in urothelial carcinoma.

## Supplementary material

Supplementary Table 1 Preclinical and clinical trials targeting DNMT in BLCA.
