# Supplementary material for: The role of DNA methylation and demethylation in bladder cancer: a focus on therapeutic strategies
Source: Front Oncol. 2025 Jun 26;15:1567242. doi: 10.3389/fonc.2025.1567242 (PMC12241037; doi:10.3389/fonc.2025.1567242)
Supplement: Supplementary Table 1 — Preclinical and clinical trials targeting DNMT in BLCA. [file Table1.docx]

| **Agent 1** | **Agent 2** | **Phase** | **Biological effect/ mechanism** | **Settings** | **Date** | **Ref.** |
| --- | --- | --- | --- | --- | --- | --- |
| 5-Aza | FK228, TSA | Preclinical | Combining 5-Aza and FK228 caused additive cytotoxicity (∼90%) in 253J, WH and UMUC3 cells. 5-Aza alone showed reduced effects on the TCC-SUP cell line, while FK228 alone was the most active. FK228 suppressed tumor growth in xenograft and orthotopic bladder cancer models. | T24, 253J, UMUC3, WH and TCC-SUP cells; xenograft | 2007 | (1) |
| 5-Aza | Sodiumphenylbutyrate | Phase I | One patient with bladder cancer showed an increase in EBV viral DNA copy number after 5-Aza treatment. While the combination of 5-AC and phenylbutyrate was well tolerated, it lacked clinical efficacy. | Locally advanced or metastatic bladder cancer | 2009 | (2) |
| 5-Aza | Cisplatin, docetaxel | Preclinical | Pre-treatment with 5-Aza increased the expression of TMS1 in wild-type and drug-resistant cells, enhancing their sensitivity to cisplatin and docetaxel. | UMUC3, TCCSUP, and T24 cells | 2011 | (3) |
| 5-Aza |  | Preclinical | Subcutaneous 5-aza showed promising clinical activity in canines.Partial remission, stable disease, and progressive disease were observed in 4/18 (22.2%), 9/18 (50.0%), and 4/18 (22.2%) dogs, respectively. Pretreatment and posttreatment methylation analysis revealed no correlation with clinical response. | Dogs with invasive urothelial carcinoma | 2012 | (4) |
| 5-Aza | TSA | Preclinical | 5-Aza (at concentrations ≥ 5 µm) and TSA (at concentrations ≥ 0.1 µm) inhibited the proliferation of canine invasive urinary cancer. | Canine invasive urinary bladder cancer | 2013 | (5) |
| 5-Aza |  | Preclinical | DNMT3A/3B was overexpressed in bladder cancer and negatively correlated with hepaCAM expression. 5-Aza downregulated DNMT3A/B expression and reversed the hypermethylation and expression of hepaCAM in bladder cancer cells. 5-Aza inhibited the proliferation and caused cell cycle arrest of bladder cancer cells. | T24, EJ and BIU-87 cells; mouse xenograft | 2016 | (6) |
| 5-Aza | Pembrolizumab, epacadostat | Phase II | Overall response rates reached 3/62 (4.8%) in patients receiving 5-Aza, pembrolizumab, and epacadostat and 1/5 (12.5%) in patients receiving 5-Aza and pembrolizumab. No data regarding bladder patients alone are available. | Advanced, metastatic solid cancers | 2020 | (7) |
| CC-486 | Carboplatin | Phase I | 3/22 patients with bladder cancer achieved PR (>94, 189, and 305 days). The disease control rate was 36.4% in bladder cancer. CC-486 was well tolerated alone or in combination with carboplatin. | Relapsed or refractory urothelial carcinoma | 2018 | (8) |
| CC-486 | - | Phase I | No results are available. | Metastatic or inoperable bladder cancer | 2018 | (9) |
| aza-T-dCyd | Decitabine | Preclinical | Aza-T-dCyd and decitabine delayed tumor growth, inhibited DNMT1 expression, and upregulated p21 levels. | Patient-derived xenograft | 2023 | (10) |
| FdCyd | THU | Phase II | Out of 1/15, bladder cancer patients achieved partial remission; the 4-month survival probability reached 42%. | Bladder cancer patients; median 3.5 prior therapy lines | 2020 | (11) |
| Decitabine | - | Preclinical | Decitabine treatment reactivated the p16 gene in cell lines not expressing p16 by demethylating the p16 promoter. In the T24 cells, p16 expression, cell growth inhibition, and G1 cell cycle arrest caused by decitabine were heritable after prolonged passage in culture. Decitabine treatment of T24 cells before injection into mice decreased the tumor growth rate. | T24, J82, and 5637 cells; nu/nu mice model | 1998 | (12) |
| Decitabine | - | Preclinical | Decitabine treatment modified the BLCA transcriptome to activate NOTCH1 and increase double-stranded RNA sensors and CK5-dependent differentiation. DAC treatment inhibited cell proliferation and caused morphological alterations reminiscent of senescence associated with IL-6 release. | HT1376, T24, B01, and B02 | 2017 | (13) |
| Decitabine | Cisplatin, gemcitabine | Preclinical | Low doses of decitabine enhanced the efficacy of cisplatin and gemcitabine on basal-like bladder cancer in vivo and in vitro. Decitabine inhibited BLCA stemness in mice, causing genome-wideDNA demethylation, gene re-expression, and changes in STAT3 signaling. | 5637 and SCaBER cells; mouse model; patient-derived xenograft | 2019 | (14) |
| Decitabine | - | Preclinical | After 8 days of treatment, decitabine induced more genes in tumorigenic cells than in nontumorigenic cells (61 vs. 34 genes). A high percentage of genes activated in both cell types belonged to the IFN signaling pathway. | T24 and LD419 cells | 2002 | (15) |
| Decitabine |  | Preclinical | Decitabine downregulated the levels of DNMT1 and DNMT3b3 proteins and their mRNA transcripts in non-dividing cells, and their mRNA transcripts were down-regulated in these cells. DNMT3a mRNA levels recovered to their original levels after drug treatment, suggesting that cell division is required for de novo methylation of CpG islands. | T24 cells | 2002 | (16) |
| Decitabine |  | Preclinical | Decitabine increased MSH3 in bladder cancer cells compared to untreated cells. | J82, T24, TCC, and UMUC cells | 2004 | (17) |
| Decitabine |  | Preclinical | Wif-1 mRNA transcript levels were inversely correlated with CpG hypermethylation of the Wif-1 promoter in bladder cancer cells. Decitabine increased Wif-1 mRNA expression in bladder tumor cell lines. | J82C, T24C, TCC, and UMUC cells | 2006 | (18) |
| Decitabine | Zabularine | Preclinical | APAF-1 methylation was found in 100% (77%) of BLCA. Compared to Decitabine, zebularine resulted in a similar prolongation of BLCA cells doubling time in T24 cells (79.8% vs. 79.6%) but not in RT4 cells (66.4% vs. 17.5%). | RT4 and T24 cells | 2006 | (19) |
| Decitabine | Cisplatin | Preclinical | Decitabine and cisplatin synergistically suppressed the growth of all BCLA cell lines independently of p53 status. Mechanistically, decitabine enhanced the ability of cisplatin to upregulate caspase activity and antiproliferative effect, increasing the number of cells in the subG1 and G2/M phases. | RT112, 253J, TCCSUP,  and T24 cells | 2008 | (20) |
| Decitabine | - | Phase I | No results are available. | Advanced solid cancers | 2008 | (21) |
| Decitabine | - | Preclinical | Decitabine decreased the growth velocity of BIU87 cells, and their survival rate decreased dose-dependently. Decitabine reversed the methylation status of  RASSF1A, while the mRNA re-expressed after treatment. | BIU87cells | 2009 | (22) |
| Decitabine | - | Preclinical | Decitabine markedly increased the levels of COL1A2 mRNA in BOY cells.  COL1A2 downregulation in BLCA occurs through CpG hypermethylation of its promoter region and may mediate BLCA proliferation and migration. | KK47, T24, UMUC, and BOY cells | 2009 | (23) |
| Decitabine | - | Preclinical | FHL1 was downregulated in BLCA through CpG hypermethylation of its promoter region, contributing to increased migration and invasion of BLCA. Decitabine increased the expression of the FHL1 mRNA transcript and markedly increased it in all tested cell lines. | T24, UMUC, and BOY cells | 2010 | (24) |
| Decitabine | - | Preclinical | The hypermethylation of the maspin promoter was observed in T24 cells. The levels of maspin mRNA and its promoter increased dose-dependent after treatment with decitabine. Decitabine inhibited proliferation, migration, and invasion and enhanced apoptosis of BLCA cells. | T24 cells | 2013 | (25) |
| Decitabine | TSA, cisplatin, gemcitabine | Preclinical | The combination of gemcitabine+cisplatin+DAC+TSA decreased the canonical Wnt/β-catenin pathway target genes, c-myc, and cyclin D1. DAC, TSA, and gemcitabine+cisplatin downregulated the expression of BCL2L1, an anti-apoptotic gene, and increased the levels of GSK3β mRNA, which in turn downregulated cyclin D1. | T24 cells | 2014 | (26) |
| Decitabine | - | Preclinical | BTG2 was hypermethylated in BLCA compared to normal urothelial tissues, and its expression was inversely correlated with the expression of DNMT1 and DNMT3a in MIBC. Decitabine upregulated BTG2 expression via the modification of CpG islands and chromatin remodeling. BTG2 upregulation reduced EJ cells tumorigenesis and invasiveness and induced G2/M arrest. | EJ cells | 2014 | (27) |
| Decitabine | - | Preclinical | CpG island in the promoter of the hepaCAM gene was hypermethylated in BLCA cells. The hypermethylation of the hepaCAM promoter was associated with its downregulation. Decitabine reversed the hypermethylation of the hepaCAM gene and increased its mRNA and protein expression. Decitabine inhibited cancer cell growth,causing cell cycle arrest at the G0/G1 phase. | T24 and BIU-87 cells | 2014 | (28) |
| Decitabine |  | Preclinical | Decitabine increased the levels of GSTM1 mRNA and protein expression. | 5637 cells | 2014 | (29) |
| Decitabine | Genistein | Phase I/II | The treatment was well tolerated, and 5/10 patients achieved stable disease for more than six months (7-14 months), including patients with thyroid carcinoma and bladder cancer. The clinical activity of this combination was modest. | Advanced solid tumors | 2015 | (30) |
| Decitabine | - | Preclinical | CBX7 is inactivated by promoter hypermethylation mediated by DNMT1 and DNMT3A in UBC. Decitabine increased CBX7 expression at mRNA and protein levels. Increased CBX7 activity suppresses BLCA progression via suppressing ERK signaling. | 5637 and UMUC-3 cells | 2021 | (31) |
| Decitabine | - | Preclinical | Hypermethylation of RSPH9 was an independent predictor of recurrence and progression of BLCA. Decitabine decreased the methylation level of RSPH9 in a dose-dependent manner. | T24 and J82 cells | 2016 | (32) |
| Decitabine | Cisplaitn, vorinostat | Preclinical | Downregulation of XOCA9 and its promoter hypermethylation were associated with aggressive disease and resistance to cisplatin in muscle-invasive BLCA. Pre-treatment with decitabine sensitized BLCA cells to cisplatin in 96-1, RT4 and SW1710 cells. Combined treatment with decitabine and vorinostat lacked efficacy in the cisplatin-resistant cell line 97-1. Vorinostat pre-treatment alone did alleviate BLCA cells' resistance to therapy. | 5637, HT1197, HT1376, J82, RT4, SCaBER, SW780, T24, TCCSUP and UM-UC3 cells | 2016 | (33) |
| Decitabine | Cisplatin, doxorubicin | Preclinical | RASSF1A was underexpressed in bladder cancer cells. Decitabine increased RASSF1A expression at mRNA and protein levels via its promoter demethylation. Decitabine induced cell cycle arrest in the G2/M phase in a dose-dependent manner and enhanced the effects of cisplatin and doxorubicin. | HT1376 and T24 cells | 2018 | (34) |
| Decitabine |  | Preclinical | Decitabine increased the median DNA methylation levels at 616 common CpGs in T24 cells in contrast to a significant decrease in the methylation level of other CpGs present in the 450K beadchip. Similar results from other cell lines indicate hypermethylation in the cancer genome may be a common response to decitabine other inhibitors or DNMT. | HCT116 and T24 cells | 2019 | (35) |
| Decitabine | Cisplatin | Preclinical | Twelve distinct CpGs in the p73 gene locus were hypermethylated BLCA compared to adjacent tissues, and low TAp73 protein expression was associated with shorter overall survival. Decitabine decreased TAp73 methylation and upregulated expression in cisplatin-resistant and wild-type T24 cells. Decitabine increased the sensitivity of both cell lines to cisplatin. | CR-T24 and T24 cells | 2019 | (36) |
| Decitabine |  | Preclinical | Decitabine increased the expression of miR-451a expression in BLCA cells. | T24 and 5637 cells | 2020 | (37) |
| Decitabine | Entinostat | Preclinical | Neither DAC nor entinostat+ cisplatin could not reverse chemoresistance. The combination of DAC and ENT synergistically induced apoptosis and cell cycle arrest in BLCA cells. This effect was selective toward cancer cells. The treatment upregulated FoxO1, activated p21, and reduced survivin expression in J82 cisplatin-resistant cells. | 82, RT-112, and HBLAK cells | 2020 | (38) |
| Decitabine | TSA | Preclinical | DAC increased vimentin levels in both cell lines. TSA alone did not change vimentin levels in TCCSUP cells. Cell treated with DAC and DAC+TSA exhibited lower levels of vimentin promoter methylation. | J82 and TCCSUP cells | 2023 | (39) |
| NTX-301 | Platinum-based chemotherapy | Phase I | No result is available. | Advanced bladder cancer; failed previous treatment | 2022 | (40) |
| Genistein | BCG | Phase II | No results are available. | Superficial bladder cancer | 2023 | (41) |
| Guadecitabine | Cisplatin, gemcitabine | Phase I | Adding guadecitabine to cisplatin and gemcitabine resulted in similar rates of severe hematological adverse events, similar cisplatin dose intensity, and modestly reduced gemcitabine dose intensity. Radical treatment options post-chemotherapy were not compromised. Guadecitabine achieved the maximal target effect at the point of cisplatin administration. No treatment-related deaths occurred. | T2-4a N0 M0 bladder urothelial carcinoma | 2021 | (42) |
| Guadecitabine | - | Preclinical | S110 caused dose-dependent genome-wide demethylation that was 5-Aza at 0.1 μmol/L and 1 μmol/L concentrations. At 10 μmol/L, only a small demethylation was noted. | T24 cells | 2007 | (43) |
| Guadecitabine | - | Preclinical | S110 was better tolerated than 5-Aza and effectively induced p16 expression in mice. After S110 treatment, reduced DNA methylation at the p16 promoter region and slowed tumor growth in human xenografts were noted. | EJ6 cells, BALB/c micexenograft | 2010 | (44) |
| Guadecitabine | Atezolizumab | Phase II | No RECIST responses were observed after therapy. Adding guadecitabine was associated with markers of immune activation and more prolonged survival. | Recurrent/advanced UC; previous progression on anti-PD-1/PD-L1 agents | 2023 | (45) |
| Zebularine |  | Preclinical | Continuous zebularine treatment caused a complete depletion of DNMT1 and partial depletion of DNMT3a and DNMT3b3. | T24 cells |  | (46) |
| Hydralazine | Procainamide | Preclinical | Hydralazine and procainamide induced de-methylation and re-expression of the ER and p16 genes in cultured cells and the re-expression of ER genes in mice.The re-expression ofthe p16 gene after hydralazine lasted longer than after 5-Aza and induced G1 arrest. | MDA-231, MCF-7, and T24 cells; BALB/c nu/nu mice xenografted with MDA-231 cells | 2003 | (47) |
| DNAzyme DT433 |  | Preclinical | DT433 caused a 50% reduction in DNMT1 methylation activity (adequate to 5-Aza activity), increasing p16 mRNA level, restoring p16 functions in a DNMT-1-dependent manner, and inhibiting T24 cell proliferation. | T24 cells | 2015 | (48) |
| SNHG1 |  | Preclinical | SNHG1 over-expression enhanced its interaction with DNMT3A and promoted its binding to the miR-129-2 promoter. The SNHG1/DNMT3A/miR-129-2-5p/Rac1 effector pathway drives the invasiveness and stemness of muscle-invasive bladder cancer. | Cell lines | 2022 | (49) |
| shDNMT3B |  | Preclinical | DNMT3B was overexpressed in BLCA. DNMT3 knockdown reduced DNMT3B knockdown reduced the methylation level of miR-451a promoters, reducing the aggressiveness of BLCA cells. | T24 and 5637 cells | 2020 | (37) |
| shDNMT3B | - | Preclinical | DNMT3B knockdown decreased methylation in the promoter of miR-34a, increased miR-34a expression, and inhibited migration, invasion, and epithelial-mesenchymal transition in BLCA cells. | T24, BIU-87, EJ, and UMUC3 cells | 2020 | (50) |
| shDNMT1 | - | Preclinical | shDNMT1 repressed DNMT1 expression and inhibited the growth and migration of BLCA cells. | T24 and J82 cells | 2020 | (51) |
| miR-152mimics | - | Preclinical | miR-152 was downregulated in BLCA. miR-152-3p mimics reduced BLCA cell viability. miR-152 inhibited the proliferation and migration of BLCA cells. | 5637, Ejm3, BIU-87, J82 and T24 cells | 2020 | (51) |
| CM-272 | Anti-PD-L1 agents | Preclinical | G9a expression was associated with poor clinical outcomes in bladder cancer. Targeting G9a/DNMT activity with CM-272 induces apoptosis and immunogenic cell death. The antitumor effect was improved by anti-PD-L1 agents, even without cisplatin. | RT112 and 5637 cells; nude mice xenograft | 2019 | (52) |
| MIL-53 | CM-272 | Preclinical | The accelerated release of CM-272 helps attenuate drug resistance in an O2 -O2-sufficient TME. Reduced glutathione levels in the TME for enhancing reactive oxygen species, ferroptosis, apoptosis, and immune cell death. | In vitro | 2023 | (53) |
| RX-3117 | - | Phase I/II | Out of 124 patients, only one completed phase I, and no completed phase II. 92 (74.2%) had disease progression. The best overall response rate in BLCA patients was 45.2. Among patients taking 700mg of RX-3117 5 times a week, only 8/80 (10%) had adverse events associated with treatment. | Metastatic bladder cancer | 2019 | (54) |

**Supplementary table 1.** Preclinical and clinical trials targeting DNMT in BLCA.

1. Karam JA, Fan J, Stanfield J, Richer E, Benaim EA, Frenkel E, et al. The use of histone deacetylase inhibitor FK228 and DNA hypomethylation agent 5-azacytidine in human bladder cancer therapy. Int J Cancer. 2007 Apr 15;120(8):1795–802.

2. Lin J, Gilbert J, Rudek MA, Zwiebel JA, Gore S, Jiemjit A, et al. A phase I dose-finding study of 5-azacytidine in combination with sodium phenylbutyrate in patients with refractory solid tumors. Clin Cancer Res. 2009 Oct 1;15(19):6241–9.

3. Ramachandran K, Gordian E, Singal R. 5-azacytidine reverses drug resistance in bladder cancer cells. Anticancer Res. 2011 Nov;31(11):3757–66.

4. Hahn NM, Bonney PL, Dhawan D, Jones DR, Balch C, Guo Z, et al. Subcutaneous 5-azacitidine treatment of naturally occurring canine urothelial carcinoma: a novel epigenetic approach to human urothelial carcinoma drug development. J Urol. 2012 Jan;187(1):302–9.

5. Dhawan D, Ramos-Vara JA, Hahn NM, Waddell J, Olbricht GR, Zheng R, et al. DNMT1: An emerging target in the treatment of invasive urinary bladder cancer. Urol Oncol. 2013 Nov 1;31(8):1761–9.

6. Wang X, Chen E, Yang X, Wang Y, Quan Z, Wu X, et al. 5-Azacytidine inhibits the proliferation of bladder cancer cells via reversal of the aberrant hypermethylation of the hepaCAM gene. Oncol Rep. 2016 Mar 1;35(3):1375–84.

7. https://clinicaltrials.gov/ct2/show/results/nct02959437 [Internet]. [cited 2024 Jan 6]. Available from: https://clinicaltrials.gov/ct2/show/results/NCT02959437

8. Von Hoff DD, Rasco DW, Heath EI, Munster PN, Schellens JHM, Isambert N, et al. Phase I Study of CC-486 Alone and in Combination with Carboplatin or nab-Paclitaxel in Patients with Relapsed or Refractory Solid Tumors. Clin Cancer Res. 2018 Sep 1;24(17):4072–80.

9. https://clinicaltrials.gov/study/nct02223052 [Internet]. [cited 2024 Jan 6]. Available from: https://clinicaltrials.gov/study/NCT02223052

10. Laranjeira ABA, Hollingshead MG, Nguyen D, Kinders RJ, Doroshow JH, Yang SX. DNA damage, demethylation and anticancer activity of DNA methyltransferase (DNMT) inhibitors. Sci Rep. 2023 Apr 12;13(1):5964.

11. Coyne GO ’sullivan, Wang L, Zlott J, Juwara L, Covey JM, Beumer JH, et al. Intravenous 5-fluoro-2’-deoxycytidine administered with tetrahydrouridine increases the proportion of p16-expressing circulating tumor cells in patients with advanced solid tumors. Cancer Chemother Pharmacol. 2020 May;85(5):979–93.

12. Bender CM, Pao MM, Jones PA. Inhibition of DNA methylation by 5-Aza-2′-deoxycytidine suppresses the growth of human tumor cell Lines1. Cancer Res. 1998 Jan 1;58(1):95–101.

13. Ramakrishnan S, Hu Q, Krishnan N, Wang D, Smit E, Granger V, et al. Decitabine, a DNA-demethylating agent, promotes differentiation via NOTCH1 signaling and alters immune-related pathways in muscle-invasive bladder cancer. Cell Death Dis. 2017 Dec 14;8(12):1–13.

14. Wu M, Sheng L, Cheng M, Zhang H, Jiang Y, Lin S, et al. Low doses of decitabine improve the chemotherapy efficacy against basal-like bladder cancer by targeting cancer stem cells. Oncogene. 2019 Jul 4;38(27):5425–39.

15. Liang G, Gonzales FA, Jones PA, Orntoft TF, Thykjaer T. Analysis of Gene Induction in Human Fibroblasts and Bladder Cancer Cells Exposed to the Methylation Inhibitor 5-Aza-2′-deoxycytidine1. Cancer Res. 2002 Feb 1;62(4):961–6.

16. Velicescu M, Weisenberger DJ, Gonzales FA, Tsai YC, Nguyen CT, Jones PA. Cell division is required for de Novo methylation of CpG islands in bladder cancer Cells1. Cancer Res. 2002 Apr 1;62(8):2378–84.

17. Kawakami T, Shiina H, Igawa M, Deguchi M, Nakajima K, Ogishima T, et al. Inactivation of the hMSH3 mismatch repair gene in bladder cancer. Biochem Biophys Res Commun. 2004 Dec 17;325(3):934–42.

18. Urakami S, Shiina H, Enokida H, Kawakami T, Tokizane T, Ogishima T, et al. Epigenetic inactivation of Wnt inhibitory factor-1 plays an important role in bladder cancer through aberrant canonical Wnt/β-catenin signaling pathway. Clin Cancer Res. 2006 Jan 15;12(2):383–91.

19. Christoph F, Kempkensteffen C, Weikert S, Köllermann J, Krause H, Miller K, et al. Methylation of tumour suppressor genes APAF-1 and DAPK-1 and in vitro effects of demethylating agents in bladder and kidney cancer. Br J Cancer. 2006 Dec;95(12):1701–7.

20. Shang D, Liu Y, Matsui Y, Ito N, Nishiyama H, Kamoto T, et al. Demethylating agent 5-Aza-2′-deoxycytidine enhances susceptibility of bladder transitional cell carcinoma to cisplatin. Urology. 2008 Jun 1;71(6):1220–5.

21. https://clinicaltrials.gov/study/nct00030615 [Internet]. [cited 2024 Jan 7]. Available from: https://clinicaltrials.gov/study/NCT00030615

22. Liu X, Dai X, Wu B. Study of 5-Aza-CdR on transcription regulation of *RASSF1A* gene in the BIU87 cell line. Urol Int. 2009;82(1):108–12.

23. Enokida. CpG hypermethylation of collagen type I α 2 contributes to proliferation and migration activity of human bladder cancer. Int J Oncol. 2009 May 7;34(6):1593–602.

24. Kawakami. CpG hypermethylation of human four-and-a-half LIM domains 1 contributes to migration and invasion activity of human bladder cancer. Int J Mol Med [Internet]. 2010 Jun 18;26(2). Available from: http://dx.doi.org/10.3892/ijmm_00000458

25. Zhang H, Qi F, Cao Y, Zu X, Chen M, Li Z, et al. 5-Aza-2′-deoxycytidine enhances *maspin* expression and inhibits proliferation, migration, and invasion of the bladder cancer T24 cell line. Cancer Biother Radiopharm. 2013 May;28(4):343–50.

26. Varol N, Konac E, Onen IH, Gurocak S, Alp E, Yilmaz A, et al. The epigenetically regulated effects of wnt antagonists on the expression of genes in the apoptosis pathway in human bladder cancer cell line (T24). DNA Cell Biol. 2014 Jul;33(7):408–17.

27. Devanand P, Kim SI, Choi YW, Sheen SS, Yim H, Ryu MS, et al. Inhibition of bladder cancer invasion by Sp1‐mediated BTG2 expression via inhibition of DNA methyltransferase 1. FEBS J. 2014 Dec;281(24):5581–601.

28. Tao J, Liu Q, Wu X, Xu X, Zhang Y, Wang Q, et al. Identification of hypermethylation in hepatocyte cell adhesion molecule gene promoter region in bladder carcinoma. Int J Med Sci. 2013;10(13):1860–7.

29. Chuang J-J, Dai Y-C, Lin Y-L, Chen Y-Y, Lin W-H, Chan H-L, et al. Downregulation of glutathione S-transferase M1 protein in N-butyl-N-(4-hydroxybutyl)nitrosamine-induced mouse bladder carcinogenesis. Toxicol Appl Pharmacol. 2014 Sep 15;279(3):322–30.

30. Kassouf E, Tehfe MA, Florescu M, Soulieres D, Lemieux B, Ayoub J-PM, et al. Phase I and II studies of the decitabine–genistein drug combination in advanced solid tumors. J Clin Oncol. 2015 May 20;33(15_suppl):e13556–e13556.

31. Huang Z, Yan Y, Zhu Z, Liu J, He X, Dalangood S, et al. CBX7 suppresses urinary bladder cancer progression via modulating AKR1B10–ERK signaling. Cell Death Dis. 2021 May 25;12(6):1–15.

32. Yoon H-Y, Kim Y-J, Kim JIS, Kim Y-W, Kang HOW, Kim WT, et al. RSPH9 methylation pattern as a prognostic indicator in patients with non-muscle invasive bladder cancer. Oncol Rep. 2016 Feb;35(2):1195–203.

33. Xylinas E, Hassler M, Zhuang D, Krzywinski M, Erdem Z, Robinson B, et al. An epigenomic approach to improving response to neoadjuvant cisplatin chemotherapy in bladder cancer. Biomolecules. 2016 Sep 2;6(3):37.

34. Khandelwal M, Anand V, Appunni S, Seth A, Singh P, Mathur S, et al. Decitabine augments cytotoxicity of cisplatin and doxorubicin to bladder cancer cells by activating hippo pathway through RASSF1A. Mol Cell Biochem. 2018 Sep;446(1–2):105–14.

35. Giri AK, Aittokallio T. DNMT inhibitors increase methylation in the cancer genome. Front Pharmacol [Internet]. 2019 Apr 24;10. Available from: http://dx.doi.org/10.3389/fphar.2019.00385

36. Bunch B, Krishnan N, Greenspan RD, Ramakrishnan S, Attwood K, Yan L, et al. TAp73 expression and P1 promoter methylation, a potential marker for chemoresponsiveness to cisplatin therapy and survival in muscle-invasive bladder cancer (MIBC). Cell Cycle. 2019 Sep 2;18(17):2055–66.

37. Liu B, Sun W, Gao W, Li L, Cao Z, Yang X, et al. microRNA-451a promoter methylation regulated by DNMT3B expedites bladder cancer development via the EPHA2/PI3K/AKT axis. BMC Cancer. 2020 Oct 21;20(1):1019.

38. Wang C, Hamacher A, Petzsch P, Köhrer K, Niegisch G, Hoffmann MJ, et al. Combination of decitabine and entinostat synergistically inhibits urothelial bladder cancer cells via activation of FoxO1. Cancers (Basel). 2020 Feb 3;12(2):337.

39. Monteiro-Reis S, Miranda-Gonçalves V, Guimarães-Teixeira C, Martins-Lima C, Lobo J, Montezuma D, et al. Vimentin epigenetic deregulation in Bladder Cancer associates with acquisition of invasive and metastatic phenotype through epithelial-to-mesenchymal transition. Int J Biol Sci. 2023 Jan 1;19(1):1–12.

40. https://clinicaltrials.gov/study/nct04851834 [Internet]. [cited 2023 Dec 31]. Available from: https://clinicaltrials.gov/study/NCT04851834

41. https://clinicaltrials.gov/study/nct01489813 [Internet]. [cited 2024 Jan 3]. Available from: https://clinicaltrials.gov/study/NCT01489813

42. Crabb SJ, Danson S, Catto JWF, Hussain S, Chan D, Dunkley D, et al. Phase I trial of DNA methyltransferase inhibitor guadecitabine combined with cisplatin and gemcitabine for solid malignancies including urothelial carcinoma (SPIRE). Clin Cancer Res. 2021 Apr 1;27(7):1882–92.

43. Yoo CB, Jeong S, Egger G, Liang G, Phiasivongsa P, Tang C, et al. Delivery of 5-Aza-2′-deoxycytidine to cells using oligodeoxynucleotides. Cancer Res. 2007 Jul 1;67(13):6400–8.

44. Chuang JC, Warner SL, Vollmer D, Vankayalapati H, Redkar S, Bearss DJ, et al. S110, a 5-Aza-2′-deoxycytidine–containing dinucleotide, is an effective DNA methylation inhibitor *in vivo* and can reduce tumor growth. Mol Cancer Ther. 2010 May 1;9(5):1443–50.

45. Jang HJ, Hostetter G, Macfarlane AW, Madaj Z, Ross EA, Hinoue T, et al. A Phase II Trial of Guadecitabine plus Atezolizumab in Metastatic Urothelial Carcinoma Progressing after Initial Immune Checkpoint Inhibitor Therapy. Clin Cancer Res. 2023 Jun 1;29(11):2052–65.

46. Cheng JC, Weisenberger DJ, Gonzales FA, Liang G, Xu G-L, Hu Y-G, et al. Continuous zebularine treatment effectively sustains demethylation in human bladder cancer cells. Mol Cell Biol. 2004 Feb;24(3):1270–8.

47. Segura-Pacheco B, Trejo-Becerril C, Perez-Cardenas E, Taja-Chayeb L, Mariscal I, Chavez A, et al. Reactivation of tumor suppressor genes by the cardiovascular drugs hydralazine and procainamide and their potential use in cancer Therapy1. Clin Cancer Res. 2003 May 1;9(5):1596–603.

48. Wang X, Zhang L, Ding N, Yang X, Zhang J, He J, et al. Identification and characterization of DNAzymes targeting DNA methyltransferase I for suppressing bladder cancer proliferation. Biochem Biophys Res Commun. 2015 May 29;461(2):329–33.

49. Xu J, Yang R, Li J, Wang L, Cohen M, Simeone DM, et al. DNMT3A/miR-129-2-5p/Rac1 Is an Effector Pathway for SNHG1 to Drive Stem-Cell-like and Invasive Behaviors of Advanced Bladder Cancer Cells. Cancers [Internet]. 2022 Aug 27;14(17). Available from: http://dx.doi.org/10.3390/cancers14174159

50. Xu K, Chen B, Li B, Li C, Zhang Y, Jiang N, et al. DNMT3B silencing suppresses migration and invasion by epigenetically promoting miR-34a in bladder cancer. Aging . 2020 Nov 20;12(23):23668–83.

51. Liu P, Wu L, Chand H, Li C, Hu X, Li Y. Silencing of miR-152 contributes to DNMT1-mediated CpG methylation of the PTEN promoter in bladder cancer. Life Sci. 2020 Nov 15;261:118311.

52. Segovia C, San José-Enériz E, Munera-Maravilla E, Martínez-Fernández M, Garate L, Miranda E, et al. Inhibition of a G9a/DNMT network triggers immune-mediated bladder cancer regression. Nat Med. 2019 Jul;25(7):1073–81.

53. Liu R, Yang J, Du Y, Yu X, Liao Y, Wang B, et al. A “One Arrow Three Eagle” Strategy to Improve CM-272 Primed Bladder Cancer Immunotherapy. Adv Mater. 2023 Dec 8;e2310522.

54. https://clinicaltrials.gov/study/nct02030067 [Internet]. [cited 2024 Jan 3]. Available from: https://clinicaltrials.gov/study/NCT02030067
